# EEG utilization to assess the efficacy of Person‐Centered Communication Intervention

**DOI:** 10.1002/alz.085673

**Published:** 2025-01-09

**Authors:** Dongseon Kim

**Affiliations:** ^1^ Sookmyung University, Seoul Korea, Republic of (South)

## Abstract

This study employs electroencephalography (EEG) to assess the efficacy of a person‐centered communication intervention in the context of dementia care. Seventy‐seven individuals in the early stage of dementia, attending day care centers, were divided into experimental and control groups following a non‐equivalence control design. The communication intervention consisted of 30 minutes of semi‐structured, topic‐specific in‐person interactions, incorporating elements of self‐introduction, rapport‐building, emotional exploration, reminiscence, and cognitive stimulation. To enhance communication with peopel with dementia, techniques such as acknowledgment, validation and other communication skills were employed, aligning with the principles of person‐centered care. Data comprises of EEG measurements on the points of prefrontal lobe before and after the intervention, and assessment of emotional changes and agitated behaviors using the Observed Emotion Rate Scale (OERS) and Agitational Behavior Scale (ABS).

Results indicate a noteworthy reduction in theta waves which are known to be associated with emotional states, alongside significant alterations in alpha and beta waves, indicative of attention increase. Moreover, statistically significant emotional changes were observed, including increased happiness and attention, as well as decreased anger, anxiety, and agitation. Notably, behavioral symptoms were found to be correlated with emotional states of anxiety and agitation, both of which exhibited reductions post‐intervention. In conclusion, the person‐centered communication intervention yielded changes in brain wave patterns and improvements in emotional and behavioral states. This study contributes to the field by providing quantitative evidence of the efficacy of person‐centered communication in alleviating anxiety and behavioral symptoms among people with dementia.

